# Antitumor activity of (*R,R’*)-4-methoxy-1-naphthylfenoterol in a rat C6 glioma xenograft model in the mouse

**DOI:** 10.1002/prp2.10

**Published:** 2013-12-05

**Authors:** Michel Bernier, Rajib K Paul, Katina S S Dossou, Artur Wnorowski, Anuradha Ramamoorthy, Arnaud Paris, Ruin Moaddel, Jean-François Cloix, Irving W Wainer

**Affiliations:** 1 Laboratory of Clinical Investigation, National Institute on Aging, National Institutes of HealthBaltimore, Maryland, 21224; 2 Institut de Chimie Organique et Analytique, ICOA, CNRS UMR7311BP6759, 45067, Orléans Cedex 2, France; 3 Laboratory of Medicinal Chemistry and Neuroengineering, Department of Chemistry, Medical University of Lublin20-093, Lublin, Poland

**Keywords:** (*R,R’*)-4-methoxy-1-naphthylfenoterol, cannabinoid receptor, glioma cell line, immunoblot analysis, microarray analysis, tumor growth, xenotransplanted tumor model

## Abstract

(*R,R’*)-4-methoxy-1-naphthylfenoterol (MNF) inhibits cancer cell proliferation in vitro through cell-type specific modulation of β2-adrenergic receptor and/or cannabinoid receptor function. Here, we report an investigation into antitumor activity of MNF in rat C6 glioma cells. The potent antiproliferative action of MNF in these cells (IC_50_ of ∼1 nmol/L) was refractory to pharmacological inhibition of β2-adrenergic receptor while a synthetic inverse agonist of cannabinoid receptor 1 significantly blocked MNF activity. The antitumor activity of MNF was then assessed in a C6 glioblastoma xenograft model in mice. Three days after subcutaneous implantation of C6 cells into the lower flank of nude mice, these animals were subjected to i.p. injections of saline or MNF (2 mg/kg) for 19 days and tumor volumes were measured over the course of the experiment. Gene expression analysis, quantitative RT-PCR and immunoblot assays were performed on the tumors after treatment. Significant reduction in mean tumor volumes was observed in mice receiving MNF when compared with the saline-treated group. We identified clusters in expression of genes involved in cellular proliferation, as well as molecular markers for glioblastoma that were significantly downregulated in tumors of MNF-treated mice as compared to saline-injected controls. The efficacy of MNF against C6 glioma cell proliferation in vivo and in vitro was accompanied by marked reduction in the expression of cell cycle regulator proteins. This study is the first demonstration of MNF-dependent chemoprevention of a glioblastoma xenograft model and may offer a potential mechanism for its anticancer action in vivo.

## Introduction

Brain glioma tumors are often lethal in human. A combination of surgery, radiation, and chemotherapy is the standard first-line therapy; however, these approaches have not significantly improved patient survival (Stupp et al. 2006) as many anticancer agents fail to cross the blood–brain barrier and/or are effluxed by ABC transporters (Agarwal et al. 2011; Haar et al. 2012). The poor outcome in treating glioma brain cancer is caused largely by various mechanisms of drug resistance and proinflammatory signaling, including increased angiogenesis, creation of a hypoxic environment, cancer stem cells, and changes in expression of microRNAs (Haar et al. 2012; Heinrich et al. 2012). The complexity of this disease necessitates the development of new therapeutic agents and strategies.

The β2-adrenergic receptor (β2-AR) has a key role in angiogenesis (Ciccarelli et al. 2011) and promotion of malignant tumor growth (Thaker et al. 2006; Wong et al. 2007), and β2-AR overexpression is frequently found in biopsies from human glioblastoma and brain tumor-associated vasculature, which is consistent with a role for β2-AR signaling during tumor vascularization (Annabi et al. 2009; Sardi et al. 2013). In addition, recent studies have demonstrated that selective β2-AR agonists impair the growth of glioma and astrocytoma cell lines through the direct stimulation of cAMP and/or associated pathways (Toll et al. 2011). Thus, the β2-AR appears to be an important new therapeutic target in the treatment of glioblastomas.

(*R,R’*)-4-methoxy-1-naphthylfenoterol (MNF) is a selective β2-AR agonist with a ∼600-fold higher affinity relative to the β1-AR and stimulates cAMP production (Jozwiak et al. 2010). We have previously characterized the antiproliferative effect of MNF in 1321N1 astrocytomas cells and demonstrated that this activity is a result of increased intracellular cAMP concentrations (Toll et al. 2011). However, when the activity of MNF, its parent compound, (*R,R’*)-fenoterol (Fen), and the prototypical β-agonist isoproterenol were tested on human HepG2 hepatocarcinoma cells, no cAMP accumulation was observed despite the detection of β2-AR (Paul et al. 2012). MNF treatment led to potent inhibition of HepG2 cell proliferation and survival while Fen and isoproterenol promoted proliferation in these cells (Paul et al. 2012). Further investigation showed the involvement of cannabinoid (CB) receptor activation in the proapoptotic action of MNF. Interestingly, the propensity of β2-AR to interact with CB receptors (Hudson et al. 2010) and other components of cellular membranes has been suggested as the source of functional differences in β2-AR signaling (Audet and Bouvier 2008).

C6 glioma is a chemically induced rat glial brain tumor cell line that possesses both β2-AR (Danner and Lohse 1997; Prenner et al. 2007) and CB receptors (Galve-Roperh et al. 2000). β-AR agonism with isoproterenol stimulates rat C6 glioma cell proliferation (Lung et al. 2005). In contrast, cannabinoids trigger apoptosis via a pathway involving CB receptor activation in C6 cells (Galve-Roperh et al. 2000; Jacobsson et al. 2001). Local administration of a synthetic CB_2_ agonist to immunocompromised mice induces regression of tumor generated by inoculation of C6 glioma cells (Sanchez et al. 2001). The growth-inhibiting actions of cannabinoids have been observed on different types of established tumor cell lines and primary cultures derived from biopsied tumor tissues, which include gliomas (Velasco et al. 2007). These data formed the basis for the use of MNF in the C6 glioblastoma model.

In this study we investigated, first, the efficacy of MNF as anti-tumor agent through modulation of β2-AR and CB receptor function in intact cells. Then, to address the importance of the cancer chemopreventive activity of MNF in vivo, we tested whether MNF administration affected tumor progression of a rat C6 glioma xenograft model in female athymic mice. Another aim of the study was to perform gene expression profiling to identify MNF target genes in order to provide a mechanistic framework for the exploration of MNF as a novel therapeutic agent for glioma tumors. Finally, because the tumor microenvironment influences how cancer cells respond to therapy (Heinrich et al. 2012; Sun et al. 2012; Curtis et al. 2013; Hayata et al. 2013), we determined whether addition of MNF to rat C6 glioma cells in culture replicated the alteration in proliferative gene expression observed in vivo.

## Materials and Methods

### Materials

(*R,R’*)-4-methoxy-1-naphthylfenoterol (MNF), and (*R,R’*)-fenoterol (Fen) were synthesized as described previously (Jozwiak et al. 2007). Dulbecco's modified Eagle Medium (DMEM), DMEM/Ham's nutrient mixture F12 (DMEM:F12, 1:1), trypsin solution, phosphate-buffered saline (PBS), fetal bovine serum (FBS), 100× solution of l-glutamine (200 mmol/L), and penicillin/streptomycin (a mixture of 10,000 units/mL penicillin and 10,000 μg/mL streptomycin) were obtained from Quality Biological (Gaithersburg, MD). The radioligands [^3^H]-Win55,212-2 (40.9 mCi/mmol) and [^3^H]-CP55,940 (144 mCi/mmol) were purchased from PerkinElmer Life Science (Boston, MA). AM251, AM630 and isoproterenol were obtained from Sigma-Aldrich (St. Louis, MO).

### Cell culture

The rat-derived C6 glioma cell line was obtained from the American Type Culture Collection (Manassas, VA). The cells were routinely maintained in DMEM supplemented with l-glutamine, 1% penicillin/streptomycin solution, and 10% FBS. CHO cells stably expressing CB1 receptors or CB2 receptors (Dossou et al. 2013) were maintained in DMEM:F12 (1:1) supplemented with l-glutamine, 2.44 g/L sodium bicarbonate, penicillin/streptomycin, and 10% FBS. All cell lines were cultured in a humidified CO_2_ incubator at 37°C.

### ^3^H-thymidine incorporation

C6 glioma cells were seeded in 24-well plates at ∼2 × 10^4^ cells/well and incubated for 24 h. Medium was replaced with serum-free DMEM and incubated with various concentrations of MNF for 24 h. Radiolabeled thymidine (10 Ci/mmol; PerkinElmer Life and Analytical Sciences, Waltham, MA) was added at 1 μCi per well for 16 h and its incorporation into DNA was then measured according to the protocol used by Paul et al. (2012). Each treatment group was performed in triplicate and two to three independent experiments were carried out.

### CB receptor ligand internalization assay

CHO cells expressing either CB1 receptors or CB2 receptors were seeded in 96-well plates at a density of ∼2 × 10^4^ cells/well for 24 h. Medium was removed and replaced with serum-free DMEM:F12 (1:1) and pretreated with increasing concentrations of MNF (0.1 nmol/L–10 μmol/L) for 1 h followed by the addition of the radioligand tracer, [^3^H]-Win55-212-2 (10 nmol/L) or [^3^H]-CP55,940 (2 nmol/L) for the determination of CB1 receptor and CB2 receptor internalization, respectively. One hour later, cells were washed twice in PBS and then lysed by the addition of 100 μL of 1.0 N NaOH. The cell-associated radioactivity was measured by liquid scintillation counting.

### Rat C6 tumor xenograft in mice

In order to assess the ability of MNF to induce regression of tumor growth in vivo, rat C6 glioma cells were trypsin-collected at confluency and were used to generate tumor xenografts. Athymic female nude mice (SWISS nu^+^/nu^+^) were obtained from Charles Rivers (L'Arbresle, France) and maintained under pathogen-free conditions with a 12 h light/12 h dark cycle. Animals were fed ad libitum with normal chow. Athymic nude mice were inoculated subcutaneously with 100 μL of culture medium containing 0.5 × 10^6^ C6 glioma cells in the left flank and then were randomly divided into two groups of 10 animals each. Starting 3 days after cell inoculation, mice received daily intraperitoneal injection (10 μL g^−1^ bw) of vehicle or MNF (2 mg kg^−1^) in 100 μmol/L ascorbic acid in saline (vehicle) 5 days a week for 19 days. Animal survival was monitored daily, and tumor size was determined with the use of a caliper to measure the length (a) and width (b) and estimated as 4/3π × rl^2^ × r^2^, where rl is the smaller and r^2^ the larger radius. The mice were monitored up to 19 days after MNF injection or euthanized earlier if the tumor size was superior to 2 cm^3^ or the mouse was lethargic, sick and unable to feed, which caused the body weight to drop below 20% of initial weight. The mice were euthanized by cervical extension, and tumor masses were removed, weighed, and washed with cold PBS before being snap frozen in liquid nitrogen. A second set of experiment with eight to nine animals in both groups was repeated. All protocols were approved by the local research ethics committee with the agreement number CLO 2009-012, and were in accordance with the European Community Council Directive of 24 November 1986 (86/609/ECC).

### Determination of MNF levels in C6 glioma tumors

The methodology can be found in Data S1.

### Analysis of gene expression in rat C6 glioma xenografts

Total RNA was isolated from rat C6 glioma xenografts harvested from vehicle and MNF-treated mice (*n* = 3 per group, cohort 1). This analysis was repeated in a second cohort of animals (*n* = 3 per group, cohort 2). Total cellular RNA was extracted using an RNeasy plus mini kit (Qiagen, Valencia, CA), and then hybridized to Illumina's Sentrix Rat Ref-12 Expression BeadChips (Illumina, San Diego, CA). Raw data were subjected to Z-normalization, as described elsewhere (Cheadle et al. 2003; Lee et al. 2012). The raw data file and the filtered, normalized results are available online in the Gene Expression Omnibus, Accession Number GSE45307 (http://www.ncbi.nlm.nih.go/geo/query/acc.cgi?acc=GSE45307). Additional information can be found in the Supplemental information.

### Total RNA extraction, cDNA synthesis and quantitative RT-PCR analysis

Total RNA (including the DNase treatment step) was isolated from frozen tumor tissues using the RNeasy mini kit (Qiagen, Valencia, CA). RNA concentration and quality was measured using the NanoDrop spectrophotometer (NanoDrop Technologies, Wilmington, DE). Subsequently, 2 μg total RNA was reverse-transcribed to cDNA using the qScript™ cDNA SuperMix (Quanta Biosciences, Gaithersburg, MD). Quantitative RT-PCR (qRT-PCR) reactions were performed to validate the expression of six genes that were selected from the microarray analysis. The reactions were carried out with SYBR® Green PCR master mix on an ABI Prism 7300 sequence detection system (Applied Biosystems) using commercially available target probes for *Sox4*, *Olig1*, *Galnt3*, *Cdkn3*, *Ccna2*, and *Bub1b* (PrimeTime qPCR Assays and Primers, IDT DNA Technologies, Coralville, IA). The genes and the catalog numbers used in this study are listed in Table S1. The data was analyzed using the 2^−ΔΔCt^ method with *Gapdh* and vehicle-treated tumors as internal controls. Controls consisting of reaction mixture without cDNA were negative in all runs.

### Western blot analysis

Frozen tumor tissues and rat C6 glioma cells were lysed with radioimmune precipitation buffer containing EGTA and EDTA (Boston BioProducts, Ashland, MA) supplemented with a phosphatase inhibitor cocktail (EMB-Calbiochem), and protease inhibitor cocktail (Sigma-Aldrich), according to standard protocols. Equal amounts of protein from the clarified lysates were separated by SDS-polyacrylamide gel electrophoresis under reducing conditions (Invitrogen, Carlsbad, CA), and electrotransferred onto polyvinylidene difluoride membranes using the iBlot system (Invitrogen). Western blots were performed according to standard methods, which involved blocking the membrane in 5% nonfat milk, followed by sequential incubation method with the primary antibody of interest and secondary antibody conjugated with the enzyme horseradish peroxidase (Paul et al. 2012). The detection of immunoreactive bands was performed by chemiluminescence using the ECL Plus Western Blotting Detection System (GE Healthcare, Piscataway, NJ). Quantitation of the protein bands was done by volume densitometry using ImageJ software (National Institutes of Health, Bethesda, MD). Primary antibodies used in this study were raised against cyclin A (sc-751, 1:500 dilution; Santa Cruz Biotechnology, Inc., Santa Cruz, CA), cyclin D1 (sc-8396; 1:500 dilution; Santa Cruz), caspase 3 (sc7148, 1:500, Santa Cruz), and β-actin (mouse; 1:10,000 dilution; Abcam, Cambridge, MA). Detection of Hsp90 with a monoclonal antibody (1:1000; Santa Cruz) was carried out to control for equal protein loading.

### Statistical analysis

Western blot data from both sets of tumor tissues were analyzed together. The Shapiro-Wilk test was used to assess if the values (protein expression levels) followed a Gaussian distribution (Shapiro and Wilk 1965). Outliers were removed from further analysis as they were preventing the population to pass the normality test. The results from rat C6 cells in culture were analyzed using the Student *t*-test. Repeated two-way analysis of variance (ANOVA) was used to compare induction of changes as a function of time. Data were expressed as means ± standard error of the mean (SEM) and were considered significant when the *P*-value was less than 0.05.

## Results

### MNF reduces tumor cell proliferation

**Figure 1:**
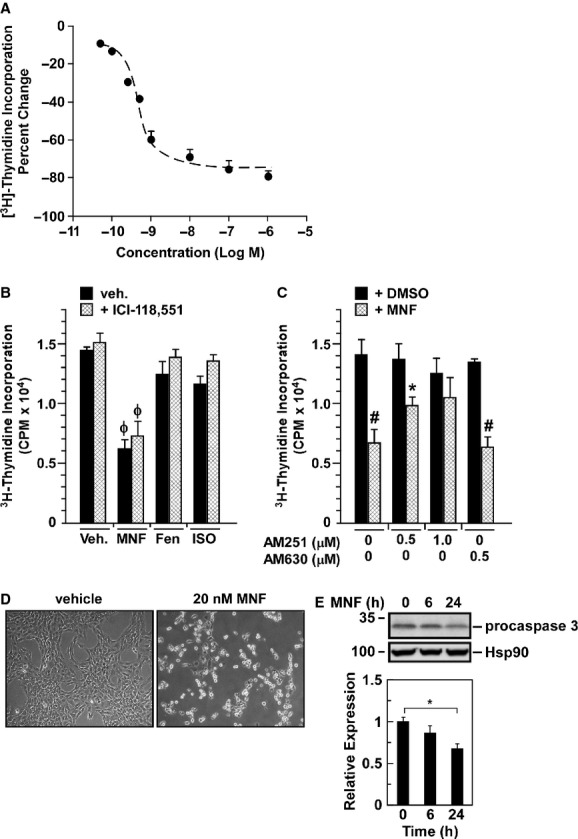
MNF reduces proliferation of rat C6 glioma cell line. A, Cell proliferation assay was performed in rat C6 glioma cells maintained in serum-free medium and treated with increasing concentrations of MNF for 24 h followed by the addition of [^3^H]-thymidine for 16 h (see Materials and Methods for further details). Data show the average ± SEM of 4 independent experiments, each performed in triplicate wells. B, C6 glioma cells in serum-free medium were pretreated without or with the selective β2-AR blocker ICI-118,551 (3 nmol/L) for 30 min followed by the addition of vehicle or 20 nmol/L of MNF, (*R,R’*)-fenoterol (Fen) or isoproterenol (ISO) for 24 h. C, C6 glioma cells were pretreated without or with the cannabinoid receptor inverse agonists AM251 (0.5 and 1 μmol/L) and AM630 (0.5 μmol/L) for 30 min followed by the addition of vehicle or 20 nmol/L of MNF for 24 h. B and C, [^3^H]-thymidine incorporation was determined after 16 h incubation. Bars represent the average ± SD of a single experiment performed in triplicate wells. Similar results were obtained in 2–3 independent experiments. *, *P* < 0.05; #, *P* < 0.01; ϕ, *P* < 0.001 versus DMSO controls. D, Changes in cell morphology were observed for C6 cells incubated with 20 nmol/L MNF for 48 h. E, C6 glioma cells were incubated with 20 nmol/L MNF for 6 and 24 h after which lysates were prepared and immunoblotted for procaspase 3. Membranes were reprobed for Hsp90, which served as a loading control. Upper panel, representative blots; lower panel, densitometric quantification of propcaspase 3 normalized to Hsp90, with the values in vehicle-treated cells set at 1.0. Bars represent means ± SEM from three independent experiments. *, *P* < 0.05.

When cell proliferation assay was performed using the rat C6 glioma cell line, a potent growth inhibition was observed in response to MNF with a IC_50_ of ∼1.0 nmol/L (Fig. 1A). The effect of the β-AR agonists isoproterenol and Fen on cell proliferation was compared to that of MNF in the absence and presence of the selective β_2_-AR blocker, ICI-118,551 (Fig. 1B). Both isoproterenol and Fen elicited weak 10–15% inhibition of C6 cell growth when used at 20 nmol/L, and the same concentration of MNF caused a significant 54.3 ± 1.2% reduction in mitogenesis (*n* = 4, *P* < 0.001). The addition of ICI-118,551 did not block the antiproliferative effect of MNF while impeding isoproterenol and Fen signaling (Fig. 1B). As indicated earlier, C6 glioma cells express both β_2_-AR and CB receptors, and the cellular actions of MNF have been reported to implicate CB receptor activity (Paul et al. 2012). Inhibition of CB1 receptors by the inverse agonist AM251 dose-dependently blocked the antiproliferative response of MNF in C6 glioma cells. In the absence of AM251, MNF reduced thymidine incorporation by more than 52.6% (*P* = 0.002) as opposed to a 28.2% (*P* = 0.011) and 16.5% (*P* = 0.159, NS) reduction when cells were pretreated with 0.5 and 1 μmol/L AM251, respectively (Fig. 1C). In contrast, inhibition of CB2 receptors with the inverse agonist AM630 had minimal effect, suggesting that the antiproliferative action of MNF occured in large part through CB1 receptor activation. Of note, a coincident change in cell morphology and nuclear condensation was observed in MNF-treated C6 glioma cells, consistent with apoptosis (Fig. 1D). Induction of apoptosis was independently confirmed by the significant reduction in procaspase-3 levels in MNF-treated cells (Fig. 1E).

**Figure 2:**
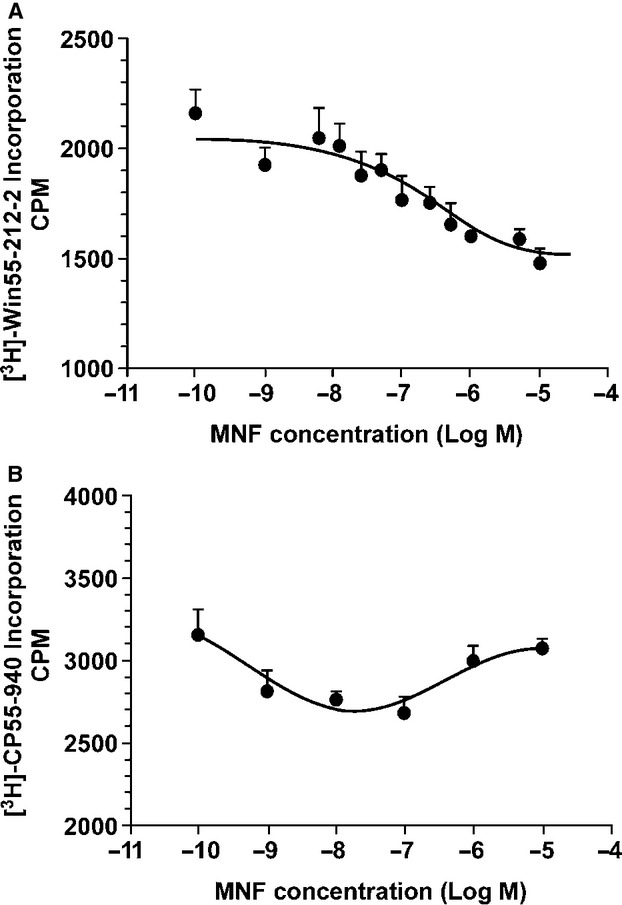
MNF exerts activity at the CB1 receptor. A, Internalization of radiolabeled CB1 receptor agonist, [^3^H]-Win55-212-2, was carried out in CHO cells stably expressing CB1 receptors. Results are presented as average ± SEM (*n* = 6) with r^2^ of 0.9025. B, Internalization of radiolabeled CB2 receptor agonist, [^3^H]-CP55-940, was carried out in CHO cells stably expressing CB2 receptors. Results are presented as average ± SEM (*n* = 6).

We have recently reported a plate-based assay that measures internalization of radiolabeled agonists for CB1 and CB2 receptors in intact CHO cells (Dossou et al. 2013). Consistent with our hypothesis, MNF was found to selectively block internalization of the CB1 receptor agonist, Win55-212-2, with an IC_50_ of 101 nmol/L (Fig. 2A). In contrast, cellular uptake of the CB2 receptor agonist, CP55-940, was refractory to MNF pretreatment in CHO/CB2 receptor cells (Fig. 2B). Thus, MNF interaction with CB1 receptor interfered, at least in part, with the binding and intracellular accumulation of a CB1 receptor agonist in CHO cells.

### MNF reduces tumor growth in vivo in a rat C6 glioma xenograft model

**Figure 3:**
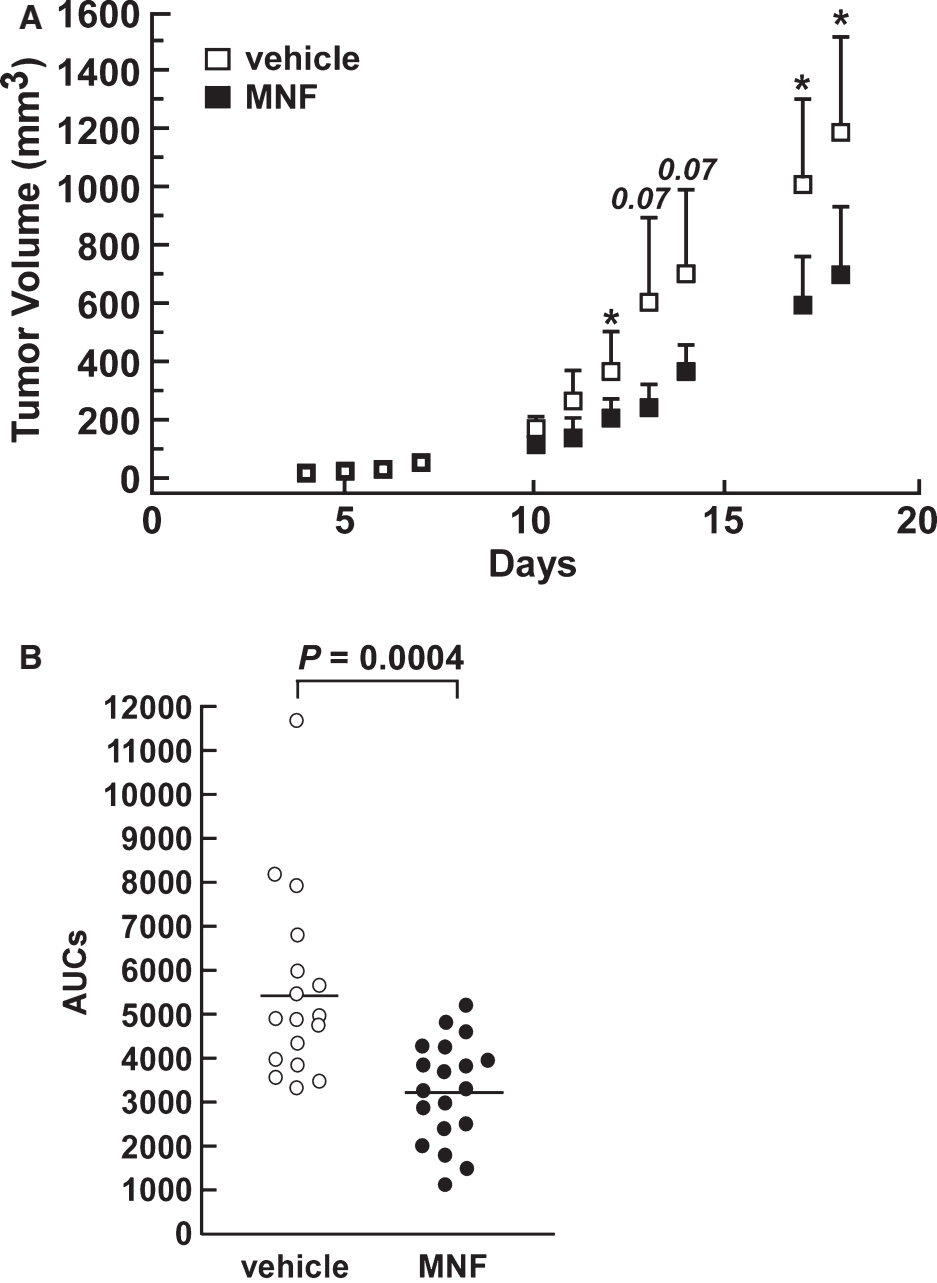
MNF reduces tumor growth in vivo in a rat C6 glioma xenograft model. C6 tumor-bearing female nude mice were assigned randomly to either the vehicle or the MNF group. Treatment was given by injecting either 2 mg/kg MNF or citrate in PBS five days a week for 18 days. Tumor volume was monitored daily and mice were sacrificed on the day after the last treatment. A, Tumor volume over time is shown for MNF-treated animals (*n* = 9) compared with vehicle-treated controls (*n* = 8). Data points represent the means ± SD. *, *P* < 0.05. Similar results were obtained in a second cohort of animals. B, Individual AUCs from the two cohorts of animals are depicted. The average AUC ± SEM for the vehicle and MNF group was 5450 ± 518 (*n* = 17) and 3217 ± 265 (*n* = 19), respectively. The *P*-value presented is for a two-tailed Student's *t*-test.

To test whether MNF might have a therapeutic effect in vivo, a rat C6 glioma xenograft model was developed in immune-deficient mice. Tumor-bearing female nude mice were treated intraperitoneally with MNF for 19 days. A modest but significant reduction in tumor volume was observed in MNF-treated animals compared with the vehicle-treated group (*P* = 0.008; Fig. 3A). These experiments were performed in a second independent cohort of mice, and showed similar results (see Fig. 3B for combined results). Tumors were excised after the last day of treatment and snap frozen in liquid nitrogen for subsequent analyses.

### Determination of MNF levels in vivo

At the completion of the study, the tumors from the MNF-treated animals were assayed for the tissue concentrations of MNF. The results indicated that significant concentrations of MNF accumulated in the tumor tissues, 141.0 ± 52.5 ng/mL per g tissue (cohort 1, *n* = 9) and 214.4 ± 65.5 ng/mL per g tissue (cohort 2, *n* = 7), demonstrating that systemically administered MNF reaches the proposed therapeutic target.

### MNF alters gene expression profiling in C6 glioma xenografts

**Figure 4:**
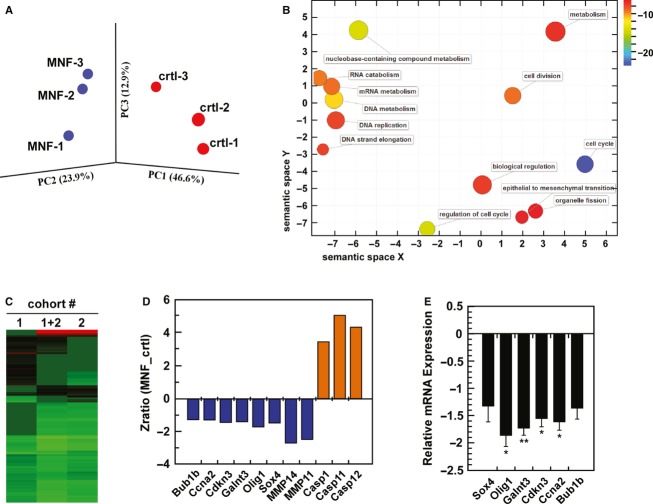
Gene expression profiling in MNF-treated C6 tumor-bearing mice. A, Gene clustering in the rat C6 xenografts: Principal component analysis (PCA) of rat C6 glioma xenograft treated with MNF vs. vehicle control. PCA was applied to the six independent samples (3 MNF, 3 controls), and numbers refer to individual sample labels. Analysis reveals clustering of samples into treatment groups. B, Visualization of enriched GO terms in a ranked list of genes that were downregulated in MNF-treated samples compared to controls. Redundant GO terms of *P*-value better than 10^−6^ were trimmed and plotted as two-dimensional scatter plot using ReviGO (see Materials and Methods for further details). Color of a GO term circle indicates the degree of enrichment according to *P*-value while its size is linked to specificity of a given GO term (more general GO terms were visualized as larger circles). Circles representing semantically similar GO terms were clustered together. C, Cluster analysis of 100 gene sets altered by MNF treatment, as compared to the control group, in cohort #1, cohort #2, and combined cohorts (1 + 2). D, Zratios of selected genes of interest is depicted, showing either up- or downregulated expression after pairwise comparison between MNF and the vehicle-treated group. E, Total RNA from C6 xenograft tumors from MNF – and vehicle-treated mice was extracted and analyzed for *Sox4*, *Olig1*, *Galnt3*, *Cdkn3*, *Ccna2* and *Bub1b* mRNA levels by quantitative RT- PCR (mean ± SD; *, *P* < 0.05; **, *P* < 0.01; *n* = 5–6). Values were normalized to GAPDH.

Global gene expression profiling by microarray analysis was then performed to identify groups of genes in C6 glioma xenografts whose expression was altered upon MNF treatment as compared to tumor-bearing vehicle-treated mice. Six independent biological samples from two cohorts of animals were used in each group. After normalization and processing of the microarray data, genes were considered as differentially expressed if they showed an absolute zratio of 1.5 or more between MNF and vehicle groups and had been assigned an adjusted *P*-value <0.05 and false discovery rate <0.3 (see Materials and Methods). Principal component analysis (PCA) revealed a discriminating pattern of significantly altered gene expression between MNF and vehicle groups (Fig. 4A). Computational analysis of the datasets derived from this experiment led to the identification of a number of cell cycle-associated GO terms, such as “DNA replication,” “Cell cycle” “Mitosis,” and “Cell division” that are likely involved in the control of tumor growth during MNF treatment (Table 1). The analysis of the overrepresented GO terms upon MNF treatment in the xenograft transcriptome revealed significant negative regulation of cell division-related genes together with those involved in control of metabolism of nucleic acids (Fig. 4B). The impact of MNF on the expression of genes implicated in the GO term “Cell cycle” (GO:0007049) can be found in Table S2. Using parameterized analysis of gene set enrichment (PAGE), we provided additional insight into regulated signaling pathways and biological processes affected by MNF. From the collection of more than 308 gene sets, there were 55 gene sets whose expression levels were significantly altered by MNF, with the majority of the gene sets (48/55) being downregulated (Table S3). The intensity of this signature from the two cohorts of animals is represented in Figure 4C and a partial list of 10 gene sets influenced most by MNF treatment is depicted in Table 2. Among the genes of interest many were downregulated in the MNF group compared to the vehicle control, including matrix metalloproteinase (MMP)-11 and 14 (Fig. 4D). Treatment with MNF also sensitized C6 glioma tumor xenograft to growth arrest via the downregulation of *Galnt3* and other cell cycle regulators, such as *Ccna2*, *Cdkn3*, and *Bub1b* (Fig. 4D). In addition, there was significant reduction in expression of Olig1 and Sox4, two molecular markers for glial brain tumors, upon MNF treatment. On the other hand, apoptosis-associated transcripts such as *Casp1*, *Casp11*, and *Casp12* were upregulated by MNF (Fig. 4D). Quantitative RT-PCR analysis confirmed that MNF decreased the expression of *Bub1b, Cdkn3*, *Ccna2*, *Olig1*, *Sox4,* and *Galnt3* as compared to control (Fig. 4E), thus validating the microarray data. Overall, these results suggest a number of possible routes by which MNF might negatively affect glioma growth and progression.

**Table 1:** List of GO Terms influenced by MNF treatment of C6 glioma in a mouse xenograft model

Annotation	GO term	Set 1	Set 2	Combined
GO0006270	DNA replication initiation	−4.0283	−3.7775	−5.0248
GO0048015	Phosphoinositide mediated signaling	−4.2781	−3.5441	−5.2545
GO0004527	Exonuclease activity	−2.5829	−5.4622	−5.8765
GO0000775	Chromosome pericentric region	−6.7644	−3.5296	−6.3081
GO0007051	Spindle organization and biogenesis	−4.6166	−5.2288	−6.7039
GO0006260	DNA replication	−4.7695	−6.0446	−7.3157
GO0007049	Cell cycle	−5.1361	−8.0162	−9.3510
GO0005634	Nucleus	−2.1680	−9.9454	−9.4291
GO0007067	Mitosis	−7.5266	−7.6563	−10.1574
GO0051301	Cell division	−6.9059	−8.0673	−10.1585

*Z* scores for ‘MNF_Crtl’ are shown. These experiments were performed on two independent cohorts of mice, with both cohorts showing equivalent results.

**Table 2:** Partial list of gene sets influenced by MNF treatment of C6 glioma in a mouse xenograft model

Size	Name	*Z* score	*P*-value	fdr
40	HIPPOCAMPUS_DEVELOPMENT_POSTNATAL	10.3511	0.0020	0.0344
280	TARTE_MATURE_PC	5.3293	0.0097	0.1073
41	HYPOPHYSECTOMY_RAT_DN	3.9294	0.0170	0.1483
30	HDACI_COLON_BUT12HRS_UP	3.6676	0.0035	0.0527
60	CELL_CYCLE	−7.4222	1.5E−10	1.73E−08
37	P21_P53_ANY_DN	−7.4300	5.12E−09	4.31E−07
74	LE_MYELIN_UP	−8.8777	6.34E−08	4.64E−06
24	CROONQUIST_IL6_STARVE_UP	−9.0059	1.87E−18	6.31E−16
65	IDX_TSA_UP_CLUSTER3	−10.1593	3.15E−20	1.76E−17
79	SERUM_FIBROBLAST_CELLCYCLE	−10.6997	2.98E−20	2.51E−17

Size indicates the number of genes found in each gene set. These experiments were performed on two independent cohorts of mice, with both cohorts showing equivalent results. Gene sets with a *Z* score >1.5 in both directions, *P* < 0.05, and false discovery rate (fdr) <0.3 are deemed significant.

**Figure 5:**
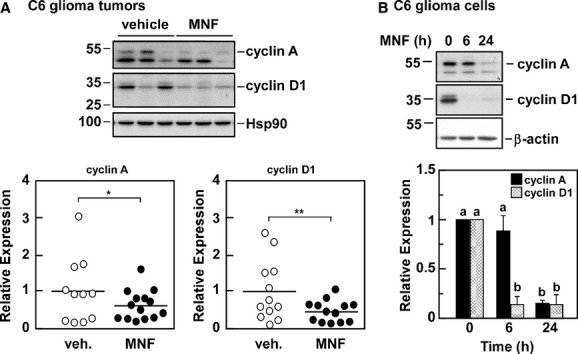
Negative impact of MNF on cyclin expression in C6 tumor xenografts. A, Lysates from tumor samples were separated by SDS-PAGE and Western blotting was carried out with primary antibodies raised against cyclin A and cyclin D1. Membranes were reprobed for Hsp90, which served as a loading control. Upper panel, representative immunoblots; lower panels, scatter plot of data showing significant difference in cyclin A and cyclin D1 expression between xenograft tumors from vehicle and MNF-treated mice, determined by densitometric quantification. *, *P* < 0.05; **, *P* < 0.01 using two-tailed Student's *t*-test. The migration of molecular-mass markers (values in kilodaltons) is shown on the left of immunoblots. B, C6 glioma cells were incubated with 20 nmol/L MNF for 6 and 24 h after which lysates were prepared and immunoblotted for cyclin A and cyclin D1. Membranes were reprobed for β-actin, which served as a loading control. Upper panel, representative blots; lower panel, densitometric quantification of cyclin A (filled bars) and cyclin D1 (hatched bars) normalized to β-actin, with the values in vehicle-treated cells set at 1.0. Bars represent means ± SEM from three independent experiments. a, b, significant difference between groups at *P* < 0.01.

C6 glioma extracts were subsequently analyzed by Western blotting with antibodies that were raised against cyclin A and cyclin D1, two key cell cycle regulators. Treatment of tumor-bearing mice with MNF led to a significant reduction in expression of both cyclins (Fig. 5A). To rule out the possibility that the tumor microenvironment influences how the C6 glioma xenograft responded to MNF, C6 glioma cells in culture were treated with 20 nmol/L MNF for 6 and 24 h, and expression changes in cyclin A and cyclin D1 were evaluated. The results indicate a significant time-dependent reduction in the expression level for both cell cycle regulators in response to MNF, whereas β-actin protein levels were not affected (Fig. 5B). In conclusion, MNF is capable of reducing growth of C6 xenografts and C6 glioma cells in culture.

## Discussion

The fenoterol analog MNF has been used by our laboratories and antiproliferative properties have been found to occur in human 1321N1 astrocytoma, HepG2 hepatocellular carcinoma and U87MG (glioblastoma-astrocytoma) cell lines after MNF treatment (Toll et al. 2011; Paul et al. 2012). Here, assessments of cellular changes in the rat C6 glioma cells have been undertaken in response to MNF and determinations of the G-coupled receptor(s) responsible for MNF signaling have been performed in this cellular model. We provide evidence that illustrates modulation of CB1 receptor activity as assessed by plate-based assay that measures internalization of a selective radiolabeled ligand. Using pharmacological inhibitors, Paul et al. 2012 recently showed the involvement of cannabinoid receptors in MNF-mediated cell growth inhibition. The current study illustrates a direct consequence of MNF interaction on CB1 receptor, which elicited reduction in cannabinoid agonist binding and intracellular accumulation. It appears unlikely, however, that the potent antiproliferative activities of MNF depend on the relatively modest inhibitory effect that MNF has on cell surface CB1 receptor internalization.

The disturbances in proliferation observed with MNF in C6 glioma cells in vitro were reproduced in C6 tumor xenografts in vivo. Our gene array study led to the identification of significantly altered relevant gene expressions and pathways. Quantitative RT-PCR and immunoblot analyses support our observations of changes in gene expressions. Dysregulated genes observed in the MNF cohort included matrix metalloproteinase (MMP)-11 and 14, which are involved in glioma growth and angiogenesis (Deryugina et al. 2002) and the development of high-grade astrocytic tumors (Stojic et al. 2008). Inhibition of MMP-2 expression has been found to reduce cultured glioma cell invasiveness (Blazquez et al. 2008). Another gene of note is *Galnt3* (UDP-GalNAc transferase 3), whose overexpression promotes cancer cell growth through its role in cell cycle regulation (Dosaka-Akita et al. 2002; Taniuchi et al. 2011).

Overexpressed in several kinds of cancers, cyclin-dependent kinase inhibitor 3 (Cdkn3) dephosphorylates and prevents CDK2 kinase activation in a cyclin-dependent manner (Hannon et al. 1994; Yeh et al. 2000; Navis et al. 2010). It has been reported that RNA levels of a major mitotic spindle assembly checkpoint gene, *Bub1b*, whose transcript encodes the mitotic checkpoint kinase MAD3L, significantly correlate with glioma grade and survival time (Bie et al. 2011). Cyclin A2 (Ccna2) is a cell cycle regulator that controls mitotic spindle orientation and cytoskeletal structures participating in the control of cell movement (Arsic et al. 2012). It is noteworthy that upregulation of Ccna2 is generally considered a marker of aggressive cancers (Yam et al. 2002). Therefore, reduction in *Cdkn3*, *Bub1b*, and *Ccna2* gene expression in the MNF-treated cohort, as compared to the control group, may represent an important antiproliferative effect of MNF.

In the past several years, the oligodendrocyte transcription factor 1 (Olig1) has been identified as a novel glioblastoma marker with diagnostic and prognostic value (Azzarelli et al. 2004; Reddy et al. 2008). Moreover, SRY-box 4 (Sox4) is a transcription factor that has been implicated in the determination of the cell fate and in tumorigenesis (reviewed by Penzo-Mendez 2010). The fact that *Olig1* and *Sox4* mRNA levels were reduced in MNF-treated C6 glioma tumors compared with the control group is consistent with decreased activation of molecular pathways leading to gliomagenesis.

The involvement of Cdkn3, Bub1b and Olig-1 in gliomagenesis is well established. Here, we provide evidence of a downregulation in the expression levels of these genes in rat C6 glioma tumor xenografts in response to MNF, suggesting that MNF and related analogs may represent a promising therapeutic strategy in the treatment of high-grade gliomas. Preliminary experiments have indicated that MNF is readily transported across the blood-brain barrier and can accumulate in the rat brain (data not shown). We are currently in the process of injecting C6 glioma cells intracranially in a syngeneic model in the rat and the effects stemming from MNF administration on survival will be reported elsewhere.

## Abbreviations

AM251: 1-(2,4-dichlorophenyl)-5-(4-iodophenyl)-4-methyl-*N*-(1-piperidyl)pyrazole-3-carboxamide
AM630: 1-[2-(morpholin-4-yl)ethyl]-2-methyl-3-(4-methoxybenzoyl)-6-iodoindole
CB: cannabinoid
Ccna2: cyclin A2
Cdkn3: cyclin-dependent kinase inhibitor 3
CHO: Chinese hamster ovary
CP55,940: 2-[(1R,2R,5R)-5-hydroxy-2-(3-hydroxypropyl) cyclohexyl]-5-(2-methyloctan-2-yl)phenol
Fen: (*R,R’*)-fenoterol
Galnt3: UDP-GalNAc transferase 3
GO: gene ontology
MMP: matrix metalloproteinase
MNF: (*R,R’*)-4-methoxy-1-naphthylfenoterol
Olig1: oligodendrocyte transcription factor 1
PAGE: parameterized analysis of gene set enrichment
PCA: principal component analysis
RT-PCR: reverse transcription polymerase chain reaction
Sox4: SRY-box 4
Win55,212-2: (R)-(+)-[2,3-dihydro-5-methyl-3-(4-morpholinylmethyl)pyrrolo[1,2,3-de]-1,4-benzoxazin-6-yl]-1-napthalenylmethanone
β2-AR: β2-adrenergic receptor

## Supporting Information

**Data S1.** Methods. **Table S1.** Catalog number and primer sequences for the qRT-PCR assays. **Table S2.** Impact of MNF treatment on the expression of genes implicated in ‘Cell cycle’ (GO:0007049). *P*-value = 9.97E−22. **Table S3.** List of gene sets significantly affected by MNF in vivo in a rat C6 glioma xenograft model in athymic mice. Significance was reached when absolute *Z* score was greater than 1.5, *P* value <0.05 and fdr <0.3.
