# Supplementary material for: Antitumor activity of (R,R’)-4-methoxy-1-naphthylfenoterol in a rat C6 glioma xenograft model in the mouse
Source: Pharmacol Res Perspect. 2013 Dec 5;1(2):e00010. doi: 10.1002/prp2.10 (PMC4186428; doi:10.1002/prp2.10)
Supplement: Supplementary file 1 [file prp20001-e00010-SD1.pdf]

SUPPLEMENTARY DATA

Antitumor Activity of (*R,R'*)-4-methoxy-1-naphthylfenoterol in a rat C6 glioma xenograft model in the mouse

Michel Bernier, Rajib K. Paul, Katina S.S. Dossou, Artur Wnorowski, Anuradha Ramamoorthy, Arnaud Paris, Ruin Moaddel, Jean-François Cloix, and Irving W. Wainer

Laboratory of Clinical Investigation National Institute on Aging, National Institutes of Health, Baltimore, Maryland 21224, USA

Supplemental Methods …………………………………………………………………… Page 2-3

Supplemental References ……………………………………………………………….. Page 4

Supplemental Table S1. **Catalog number and primer sequences for the qRT-PCR assays**….Page 5

Supplemental Table S2. **Impact of MNF treatment of the expression of genes implicated in 'Cell Cycle' (GO:0007049)**……………………………………………………………… Page 6-8

Supplemental Table S3. **List of gene sets significantly affected by MNF *in vivo* in a rat C6 glioma xenograft model in athymic mice**…………………………………………………… Page 9-10

Supplemental Methods

Determination of MNF levels in C6 glioma tumours. The accumulation of MNF *in vivo* in C6 tumour xenografts in athymic mice was assessed in comparison with vehicle-treated tumour-bearing animals. The frozen tumour samples were thawed, and 300-400 mg tumour tissue was placed in 300 µl of water supplemented with 9 µl acetonitrile containing 500 ng/ml of methoxyfenoterol as the internal standard. After thorough homogeneization of the tumour tissue on ice using a Polytron micro homogenizer (Pro200 Bio-gen series, Proscientific , Oxford, CT, USA), 600 µl acetonitrile was added and the mixture was vortexed for 10 s. The solution was centrifuged at 20,800 rcf for 10 min at 4ºC. The pellet was discarded and the supernatant was centrifuged again for an additional 10 min before being transferred to a Shimadzu SIL-20A autosampler for analysis. The samples were maintained in the autosampler tray at 4ºC, and injections of 20 µl aliquots were made to an analytical HPLC column as followed:

The separation of MNF was accomplished by HPLC (Shimadzu Prominence HPLC system; Shimadzu, Columbia, MD, USA) followed by LC-MS/MS. In brief, the assays were conducted using an Eclipse XDB-C_{18} guard column (4.6 mm x 12.5 mm) and an Atlantis HILIC analytical column (150 x 2.1 mm ID, 5 µm). The mobile phase consisted of water containing 0.1% formic acid as component A and acetonitrile as component B. A linear gradient was run as follows: 0 min 95% B; 5 min 60% B; 6 min 80% B; 10 min 95% B at a flow rate of 1.0 ml/min. The total run time was 20 min per sample. The calibration curve was prepared in a similar fashion as the samples, with 300-400 mg of tumor tissue spiked with a 0.5 serial dilution of MNF (300 to 9.375 ng/ml) and the addition of 9 µl acetonitrile containing 500 ng/ml of methoxyfenoterol.

Identification and quantification of the analytes was accomplished using a triple quadrupole API-4000 LC-MS/MS system equipped with Turbo Ion Spray® (TIS) (Applied Biosystems, Foster City, CA, USA). The data, which was acquired in positive electrospray ionization mode and multiple reaction monitoring (MRM), was analyzed with Analyst v.1.4.2 (Applied Biosystems). The standards were characterized using the following MRM transitions: MNF (369-200) and methoxyfenoterol (318-149) as the internal standard. Tumor tissues from vehicle-injected mice were used as negative controls. The TIS instrumental source settings for temperature, curtain gas, ion source gas 1 (nebulizer), ion source gas 2 (turbo ion spray), entrance potential and ion spray voltage were 500 °C, 10 psi, 60 psi, 60 psi, 10V and 5500 V, respectively. The TIS compound parameter settings for declustering potential, collision energy, and collision cell exit potential were 70V, 30V, 12V for MNF; 95V, 26V and 12V for methoxyfenoterol

Analysis of gene expression in rat C6 glioma xenografts. Total cellular RNA from rat C6 glioma xenografts was extracted using an RNeasy plus mini kit (QIAGEN, Valencia, CA, USA), and its quality

was assessed using an Agilent BioAnalyzer using RNA 6000 Nano Chips (Agilent Technologies, Santa Clara, CA, USA). Transcriptional profiling was determined using Illumina Sentrix BeadChips (Illumina, San Diego, CA, USA). Total RNA was used to generate biotin-labeled cRNA with the Illumina TotalPrep RNA Amplification Kit. In short, 0.5ug of total RNA was first converted into single-stranded cDNA with reverse transcriptase using an oligo-dT primer containing the T7 RNA polymerase promoter site and then copied to produce double-stranded cDNA molecules. The double-stranded cDNA was cleaned and concentrated with the supplied columns and used in an overnight in-vitro transcription reaction where single-stranded RNA (cRNA) was generated incorporating biotin-16-UTP. A total of 0.75µg of biotin-labeled cRNA was hybridized at 58 °C for 16 h to Illumina's Sentrix Rat Ref-12 Expression BeadChips. Each BeadChip has ~22,000 well-annotated RefSeq transcripts with approximately 30-fold redundancy. The arrays were washed, blocked and the labeled cRNA was detected by staining with streptavidin-Cy3. Hybridized arrays were scanned using an Illumina BeadStation 500X Genetic Analysis Systems scanner and the image data extracted using Illumina's GenomeStudio software, version 1.6.1. For statistical analysis, the expression data were filtered to include only probes with a consistent signal on each chip and an Illumina detecton p value < 0.02.

Correlation analysis, sample clustering analysis and principal component analysis was performed to identify/exclude any possible outliers. The resulting dataset was next analyzed with DIANE 6.0, a spreadsheet-based microarray analysis program using value statistics for Z-Score reliability below 0.05; and mean background-corrected signal intensity greater than zero (www.grc.nia.nih.gov/branches/rrb/dna/diane_software.pdf). A minimum hypergeometric algorithm embedded into GOrilla web-based application (http://cbl-gorilla.cs.technion.ac.il) was used to identify enriched gene ontology (GO) terms in a ranked list of genes (Eden *et al,* 2009). ReviGO, a semantic similarity software available at http://revigo.irb.hr, was utilized to reduce the redundancy and to visualize the overrepresented GO terms (Supek *et al,* 2011).

Gene set enrichment analysis use gene expression values or gene expression change values for all of the genes in the microarray. Parametric analysis of gene set enrichment (PAGE) was used using the WEB-*PAGE* GSA tool (De *et al,* 2010) for gene set analysis. Gene Sets include the MSIG database [Link], Gene Ontology Database [Link], GAD human disease and mouse phenotype gene sets (Zhang *et al,* 2010) were used to explore functional level changes. Gene-gene interaction was also analyzed using the Ingenuity Pathway Analysis (IPA) system (Ingenuity® Systems, www.ingenuity.com).

Supplemental References

De S, Zhang Y, Garner JR, Wang SA, Becker KG (2010) Disease and phenotype gene set analysis of disease-based gene expression in mouse and human. *Physiol Genomics* **42A**: 162-167

Eden E, Navon R, Steinfeld I, Lipson D, Yakhini Z (2009) GOrilla: a tool for discovery and visualization of enriched GO terms in ranked gene lists. *BMC Bioinformatics* **10**:48

Supek F, Bosnjak M, Skunca N, Smuc T (2011) REVIGO summarizes and visualizes long lists of gene ontology terms. *PLoS One* **6**: e21800

Zhang Y, De S, Garner JR, Smith K, Wang SA, Becker KG (2010) Systematic analysis, comparison, and integration of disease based human genetic association data and mouse genetic phenotypic information. *BMC Med Genomics* **3**: 1

Supplementary Table S1. Catalog number and primer sequences for the qRT-PCR assays.

Gene	Catalog No.	Primer Sequence (5' ->3')
Sox4	Rn.PT.53a.6333566.g	CAC CAG TTT CCA CCT TTC AAC
		TCT AGA CAG AAC AGC AAT TCG T
Olig1	Rn.PT.53a.5294009.g	GCC ACG AGT ACA AAC ATC AAG
		GAA CTT CTC CAC TCC GAA ACC
Galnt3	Rn.PT.53a.34846667	CTT CTG GAT GTT GTG TCG GAT
		TTT ATG TCT GGA TGT CGG TGA G
Cdkn3	Rn.PT.53a.34657684	CAC ATA AAC CGA GAA ACT GAG AAC
		CCG CCC ATT TCA ATA CAA GC
Ccna2	Rn.PT.53a.10705752	CAT TCA CTG GCT TTT CGT CTT C
		CTT TTA GTG CCG CTG TCT CT
Bub1b	Rn.PT.53a.11858243	CTG ATG ACT CTG AAC TCT CTG C
		TCC CAA CTT TAC TCC GTA TGT G
Gapdh	Rn.PT.53a.37055410	GTA ACC AGG CGT CCG ATA C
		GTT CTA GAG ACA GCC GCA TC

Supplemental Table S2. Impact of MNF treatment on the expression of genes implicated in 'Cell cycle' (GO:0007049). P-value = 9.97E-22.

Symbol	Full name	Fold change MNF:crtl
KIF20A	kinesin family member 20a	-1.44
CDC20	cell division cycle 20 homolog (s. cerevisiae)	-1.42
FBXO5	f-box protein 5	-1.42
KIF23	kinesin family member 23	-1.42
CCNB2	cyclin b2	-1.39
AURKB	aurora kinase b	-1.37
RFC4	replication factor c (activator 1) 4, 37kda	-1.36
DCTN3	dynactin 3 (p22)	-1.33
PFDN1	prefoldin subunit 1	-1.31
PLK1	polo-like kinase 1	-1.31
MCM6	minichromosome maintenance complex component 6	-1.30
UBE2C	ubiquitin-conjugating enzyme e2c	-1.30
BUB1B	budding uninhibited by benzimidazoles 1 homolog beta (yeast)	-1.29
CCNA2	cyclin a2	-1.28
POLE2	polymerase (dna directed), epsilon 2, accessory subunit	-1.28
PSMB6	proteasome (prosome, macropain) subunit, beta type, 6	-1.28
GMNN	geminin, dna replication inhibitor	-1.27
RALA	v-ral simian leukemia viral oncogene homolog a (ras related)	-1.26
E2F8	e2f transcription factor 8	-1.25
PSMB9	proteasome (prosome, macropain) subunit, beta type, 9	-1.25
GINS4	gins complex subunit 4 (sld5 homolog)	-1.24
SUV39H1	suppressor of variegation 3-9 homolog 1 (drosophila)	-1.24
CDCA8	cell division cycle associated 8	-1.23
GADD45GIP1	growth arrest and dna-damage-inducible, gamma interacting protein 1	-1.23
PLK4	polo-like kinase 4	-1.23
CDC6	cell division cycle 6 homolog (s. cerevisiae)	-1.22
POLD1	polymerase (dna directed), delta 1, catalytic subunit 125kda	-1.22
PSMB8	proteasome (prosome, macropain) subunit, beta type, 8	-1.22
ANXA11	annexin a11	-1.20
KNTC1	kinetochore associated 1	-1.20
RRM2	ribonucleotide reductase m2	-1.20
KIF2C	kinesin family member 2c	-1.19
NASP	nuclear autoantigenic sperm protein (histone-binding)	-1.19
PSMA5	proteasome (prosome, macropain) subunit, alpha type, 5	-1.19
PSMB10	proteasome (prosome, macropain) subunit, beta type, 10	-1.19
LIG1	ligase i, dna, atp-dependent	-1.18
MYBL2	v-myb myeloblastosis viral oncogene homolog (avian)-like 2	-1.18
NUP107	nucleoporin 107kda	-1.18
POLD2	polymerase (dna directed), delta 2, accessory subunit	-1.18
CENPE	centromere protein e, 312kda	-1.17

CENPF	centromere protein f, 350/400kda (mitosin)	-1.17
MCM3	minichromosome maintenance complex component 3	-1.17
CENPL	centromere protein l	-1.16
CHAF1B	chromatin assembly factor 1, subunit b (p60)	-1.16
GNAI2	guanine nucleotide binding protein (g protein), αi2	-1.16
MCM7	minichromosome maintenance complex component 7	-1.16
NUF2	nuf2, ndc80 kinetochore complex component, homolog (s. cerevisiae)	-1.16
TUBG1	tubulin, gamma 1	-1.16
BCCIP	brca2 and cdkn1a interacting protein	-1.15
MCM2	minichromosome maintenance complex component 2	-1.15
PKMYT1	protein kinase, membrane associated tyrosine/threonine 1	-1.15
POLA1	polymerase (dna directed), alpha 1, catalytic subunit	-1.15
POLA2	polymerase (dna directed), alpha 2, accessory subunit	-1.15
SIAH2	siah e3 ubiquitin protein ligase 2	-1.15
BANP	btg3 associated nuclear protein	-1.14
CEP76	centrosomal protein 76kda	-1.14
PCNA	proliferating cell nuclear antigen	-1.14
PRIM1	primase, dna, polypeptide 1 (49kda)	-1.14
BRD7	bromodomain containing 7	-1.13
CDK6	cyclin-dependent kinase 6	-1.13
DYNC1I2	dynein, cytoplasmic 1, intermediate chain 2	-1.13
FOXM1	forkhead box m1	-1.13
PSMC3	proteasome (prosome, macropain) 26s subunit, atpase, 3	-1.13
CDK9	cyclin-dependent kinase 9	-1.12
MCM8	minichromosome maintenance complex component 8	-1.12
MNAT1	menage a trois homolog 1, cyclin h assembly factor (xenopus laevis)	-1.12
NUP85	nucleoporin 85kda	-1.12
ODF2	outer dense fiber of sperm tails 2	-1.12
CENPT	centromere protein t	-1.11
MCM10	minichromosome maintenance complex component 10	-1.11
MCM5	minichromosome maintenance complex component 5	-1.11
RGS2	regulator of g-protein signaling 2, 24kda	-1.11
USP3	ubiquitin specific peptidase 3	-1.11
ANAPC1	anaphase promoting complex subunit 1	-1.10
CDKN2C	cyclin-dependent kinase inhibitor 2c (p18, inhibits cdk4)	-1.10
DCTN1	dynactin 1	-1.10
ERF	ets2 repressor factor	-1.10
MCTS1	malignant t cell amplified sequence 1	-1.10
PKN2	protein kinase n2	-1.10
CUL1	cullin 1	-1.09
PHF13	phd finger protein 13	-1.09
ZMYND11	zinc finger, mynd-type containing 11	-1.09
BTRC	beta-transducin repeat containing e3 ubiquitin protein ligase	-1.07
MAPRE1	microtubule-associated protein, rp/eb family, member 1	-1.07
UHRF2	ubiquitin-like with phd and ring finger domains 2, e3 ubiquitin ligase	-1.07
CDC25B	cell division cycle 25 homolog b (s. pombe)	-1.06

MAPK6	mitogen-activated protein kinase 6	-1.04
PSMD11	proteasome (prosome, macropain) 26s subunit, non-atpase, 11	-1.04
MAPK1	mitogen-activated protein kinase 1	-1.01
SPIN1	spindlin 1	-1.00
CSNK2A2	casein kinase 2, alpha prime polypeptide	+1.01
XPO1	exportin 1 (crm1 homolog, yeast)	+1.01
NDEL1	nude nuclear distribution e homolog (a. nidulans)-like 1	+1.02
HSP90AA1	heat shock protein 90kda alpha (cytosolic), class a member 1	+1.03
DUSP1	dual specificity phosphatase 1	+1.28

Supplemental Table S3. List of gene sets significantly affected by MNF *in vivo* in a rat C6 glioma xenograft model in athymic mice. Significance was reached when absolute Zscore was greater than 1.5, *P* value < 0.05 and fdr < 0.3.

	MNF_Control, cohort 1			MNF_Control, cohort 2			MNF_Control, combined cohorts		
**PathwayName**	**(Zscore)**	**(P_value)**	**(fdr)**	**(Zscore)**	**(P_value)**	**(fdr)**	**(Zscore)**	**(P_value)**	**(fdr)**
HIPPOCAMPUS_DEVELOPMENT_POSTNATAL	3.6731	0.0211	0.1456	10.3512	0.0020	0.0344	11.4209	0.0017	0.0282
TARTE_MATURE_PC	3.6636	0.0129	0.1088	5.3293	0.0097	0.1073	6.8026	0.0004	0.0085
HDACI_COLON_BUT12HRS_UP	5.3212	0.0002	0.0053	3.6676	0.0035	0.0527	5.8481	0.0000	0.0007
HYPOPHYSECTOMY_RAT_DN	3.6187	0.0459	0.2249	3.9294	0.0170	0.1483	5.3951	0.0008	0.0144
HDACI_COLON_TSABUT_UP	4.1465	0.0080	0.0751	3.4480	0.0042	0.0600	5.2313	0.0006	0.0109
HDACI_COLON_BUT16HRS_UP	3.6723	0.0199	0.1426	3.4287	0.0072	0.0877	4.8024	0.0010	0.0176
HDACI_COLON_BUT24HRS_UP	3.0864	0.0323	0.1878	3.3154	0.0119	0.1209	4.4492	0.0010	0.0169
ATRBRCAPATHWAY	-1.8815	0.0406	0.2138	-2.9977	0.0000	0.0000	-3.4407	0.0000	0.0002
GOLDRATH_HP	-2.4433	0.0229	0.1518	-2.2875	0.0441	0.2766	-3.4750	0.0007	0.0141
BRENTANI_REPAIR	-3.8568	0.0002	0.0052	-2.9453	0.0101	0.1094	-4.2370	0.0002	0.0048
REN_E2F1_TARGETS	-2.9150	0.0121	0.1044	-3.5319	0.0026	0.0414	-4.4393	0.0008	0.0145
MOREAUX_TACI_HI_IN_PPC_UP	-2.9881	0.0064	0.0635	-3.8456	0.0007	0.0154	-4.9180	0.0000	0.0009
LAMB_CYCLIN_D3_GLOCUS	-2.8637	0.0360	0.2028	-4.3054	0.0000	0.0000	-5.0503	0.0000	0.0001
POD1_KO_UP	-3.9363	0.0052	0.0553	-4.3565	0.0064	0.0812	-5.1416	0.0007	0.0129
MOREAUX_TACI_HI_VS_LOW_DN	-4.3721	0.0000	0.0011	-3.2936	0.0020	0.0334	-5.1872	0.0000	0.0000
E2F1_DNA_UP	-3.6781	0.0008	0.0142	-4.4885	0.0004	0.0099	-5.4288	0.0000	0.0014
SHEPARD_CRASH_AND_BURN_MUT_VS_WT_DN	-3.9994	0.0035	0.0413	-4.0540	0.0139	0.1315	-5.5788	0.0009	0.0160
IRITANI_ADPROX_LYMPH	-5.1468	0.0007	0.0132	-3.3728	0.0387	0.2504	-5.7142	0.0004	0.0075
SHEPARD_GENES_COMMON_BW_CB_MO	-4.0300	0.0067	0.0656	-4.3867	0.0259	0.1927	-5.7440	0.0019	0.0308
PEART_HISTONE_DN	-2.5055	0.0408	0.2142	-5.2170	0.0002	0.0063	-5.7817	0.0001	0.0017
SHEPARD_BMYB_MORPHOLINO_DN	-4.3350	0.0022	0.0300	-4.7947	0.0100	0.1089	-6.0639	0.0002	0.0054
DAC_FIBRO_DN	-4.4476	0.0007	0.0127	-4.9523	0.0007	0.0142	-6.4794	0.0000	0.0002
BRCA_PROGNOSIS_NEG	-3.2974	0.0377	0.2052	-5.4682	0.0000	0.0003	-6.5976	0.0000	0.0002
MANALO_HYPOXIA_DN	-3.2723	0.0019	0.0268	-6.1956	0.0000	0.0000	-6.7155	0.0000	0.0000
LEE_TCELLS2_UP	-4.1051	0.0020	0.0282	-5.6671	0.0000	0.0006	-6.9464	0.0000	0.0000
KENNY_WNT_UP	-4.0135	0.0093	0.0858	-5.5586	0.0000	0.0000	-6.9481	0.0000	0.0000
STEMCELL_NEURAL_UP	-6.0983	0.0000	0.0001	-5.0064	0.0000	0.0010	-7.2833	0.0000	0.0000
SASAKI_ATL_UP	-5.0963	0.0000	0.0010	-5.5299	0.0000	0.0005	-7.2897	0.0000	0.0000
SASAKI_TCELL_LYMPHOMA_VS_CD4_UP	-5.0963	0.0000	0.0010	-5.5299	0.0000	0.0005	-7.2897	0.0000	0.0000
DNA_REPLICATION_REACTOME	-3.8683	0.0039	0.0450	-6.5700	0.0000	0.0000	-7.3185	0.0000	0.0000
VERNELL_PRB_CLSTR1	-3.4691	0.0269	0.1692	-6.5636	0.0000	0.0000	-7.4144	0.0000	0.0000
GAY_YY1_DN	-7.6370	0.0000	0.0000	-4.7456	0.0016	0.0295	-7.4972	0.0000	0.0001
CANCER_UNDIFFERENTIATED_META_UP	-4.8288	0.0001	0.0034	-6.2847	0.0000	0.0013	-7.7981	0.0000	0.0002
CELL_CYCLE_KEGG	-4.1925	0.0008	0.0139	-7.0955	0.0000	0.0000	-7.8764	0.0000	0.0000
ADIP_DIFF_CLUSTER5	-4.6054	0.0050	0.0547	-6.8850	0.0000	0.0006	-7.9684	0.0000	0.0005
OLDAGE_DN	-4.5391	0.0006	0.0115	-7.1043	0.0000	0.0001	-8.1220	0.0000	0.0000
MIDDLEAGE_DN	-3.6696	0.0102	0.0919	-7.7600	0.0000	0.0000	-8.2457	0.0000	0.0000
TARTE_PLASMA_BLASTIC	-4.8905	0.0002	0.0054	-6.7905	0.0000	0.0000	-8.3308	0.0000	0.0000
YU_CMYC_UP	-4.4689	0.0049	0.0539	-7.3723	0.0000	0.0000	-8.4501	0.0000	0.0000
CMV_IE86_UP	-6.1497	0.0000	0.0000	-6.5954	0.0000	0.0000	-8.5032	0.0000	0.0000
HOFFMANN_BIVSBII_BI_TABLE2	-5.1273	0.0001	0.0030	-7.7114	0.0000	0.0005	-8.5773	0.0000	0.0002

CELL_CYCLE	-5.0187	0.0000	0.0013	-7.4222	0.0000	0.0000	-8.6730	0.0000	0.0000
KAMMINGA_EZH2_TARGETS	-5.3211	0.0000	0.0004	-7.2448	0.0000	0.0000	-8.8163	0.0000	0.0000
P21_P53_ANY_DN	-5.0275	0.0012	0.0186	-7.4300	0.0000	0.0000	-8.8530	0.0000	0.0000
PRMT5_KD_UP	-9.3903	0.0000	0.0000	-5.4620	0.0000	0.0010	-8.9362	0.0000	0.0000
CROONQUIST_IL6_RAS_DN	-6.7147	0.0000	0.0000	-7.7040	0.0000	0.0000	-9.7453	0.0000	0.0000
DOX_RESIST_GASTRIC_UP	-5.8557	0.0000	0.0001	-8.6145	0.0000	0.0000	-10.1840	0.0000	0.0000
LEE_TCELLS3_UP	-7.2409	0.0000	0.0000	-8.0339	0.0000	0.0000	-10.3427	0.0000	0.0000
LI_FETAL_VS_WT_KIDNEY_DN	-5.4447	0.0001	0.0024	-9.1715	0.0000	0.0000	-10.4609	0.0000	0.0000
ZHAN_MM_CD138_PR_VS_REST	-6.1404	0.0000	0.0000	-9.0080	0.0000	0.0000	-10.7002	0.0000	0.0000
CROONQUIST_IL6_STARVE_UP	-6.5055	0.0000	0.0000	-9.0059	0.0000	0.0000	-10.7172	0.0000	0.0000
LE_MYELIN_UP	-6.5477	0.0000	0.0009	-8.8777	0.0000	0.0000	-10.8010	0.0000	0.0000
IDX_TSA_UP_CLUSTER3	-6.1057	0.0000	0.0008	-10.1593	0.0000	0.0000	-11.3388	0.0000	0.0000
SERUM_FIBROBLAST_CELLCYCLE	-7.2985	0.0000	0.0000	-10.6997	0.0000	0.0000	-12.2668	0.0000	0.0000
